# Screening obstructive sleep apnea-hypopnea syndrome in hypertensive patients: a comparative study of the efficiency of the Epworth sleepiness scale

**DOI:** 10.1186/s12890-018-0737-y

**Published:** 2018-11-21

**Authors:** Florent Seguro, Vincent Bard, Kamila Sedkaoui, Maya Riche, Alain Didier, Béatrice Bouhanick

**Affiliations:** 1 Department of Therapeutics and Hypertension, TSA 50032, Rangueil UniversityHospital, 31059 cedex 9 Toulouse, France; 2 Department of Pneumology, TSA 30030, Toulouse Hospital University, 31059 cedex 9 Toulouse, France; 3 Cardiology Unit, Clinique de l’Union, Saint Jean, 31242 Toulouse, France; 4 UMR 1027 INSERM Toulouse 3 University, Toulouse, France

**Keywords:** Obstructive sleep apnea Hypnopnea syndrome, Hypertension, Epworth sleepiness scale

## Abstract

**Background:**

Untreated Obstructive Sleep Apnea Hypnopnea Syndrome (OSAHS) is a known factor contributing to resistant hypertension (HT). Continuous Positive Airways Pressure (CPAP) is effective to decrease blood pressure (BP) in severe OSAHS. In our clinical practice, hypertensive patients seem less symptomatic with regard to severe OSAHS than normotensive patients, leading to a risk of underdiagnosis when OSAHS is screened with Epworth Sleepiness Scale (ESS). We aimed to confirm that severe OSAHS is less symptomatic in HT patients than normotensive patients using ESS.

**Methods:**

We retrospectively compared two age, gender-matched groups - 100 hypertensive patients and 100 normotensive patients - with severe OSAHS defined as an AHI (Apnea Hypopnea Index) ≥30. OSAHS was considered symptomatic when ESS > 10.

**Results:**

The two groups of patients did not differ significantly with respect to main characteristics including Body Mass Index (BMI), AHI and ODI (Oxygen Desaturation Index). Systolic and Diastolic BP were higher in HT patients (*p* < 0.01). HT patients were less symptomatic with regard to severe OSAHS with a lower ESS (10.0 vs 11.9, *p* < 0.01), and a lower number of patients with an ESS > 10 (30% vs 58%, p < 0.01). In multivariable analysis adjusted on age, gender, Obesity, Systolic BP, Diastolic BP, AHI and ADO, normotension was significantly associated with symptomatic OSAHS (OR = 2.83, [1.298–6.192], p < 0.01).

**Conclusions:**

In our study on patients with severe OSAHS, ESS score was lower in hypertensive patients than in normotensive patients. This discrepancy may lead to an underestimation of severe OSAHS in hypertensive patients.

## Introduction

Obstructive Sleep Apnea Hypnopnea Syndrome (OSAHS) is widely acknowledged as a common chronic pathology, but to date it has remained under-diagnosed [1, 2]. OSAHS is often associated with multiple co-morbidities including hypertension [3] (HT) which is the most common cardiovascular disease in adults [4]. It is estimated that more than half the cases with OSAHS are hypertensive [5]s, and that 30% of hypertensive patients have OSAHS [6].

It is now recognized amongst scientific circles that OSAHS constitutes a major risk factor for both elevated blood pressure [7] and for developing resistant HT [8]. Several studies have demonstrated that hypertensive patients with OSAHS have a higher risk of developing cardiovascular events than those without it [9–11], and that Continuous Positive Airway Pressure therapy (CPAP) improves blood pressure control in compliant patients (≥ 4 h per night) [12, 13]. However, the real benefit of such a treatment as regards cardiovascular-related mortality is still uncertain [14].

Nocturnal polysomnography is the one standard examination for diagnosing OSAHS [15]. Because of its limited accessibility, questionnaires have been developed to assess excessive daytime sleepiness, which turned out to be a major symptom of OSAHS. Excessive daytime sleepiness is also used as a criterion for the screening of OSAHS not only by the American Academy of Sleep Medicine (AASM) [16] but also by the joint recommendations of the European Society of Hypertension and the European Respiratory Society [17].

It follows from the above guidelines [16, 17], that the measurement of this daytime sleepiness can be subjectively evaluated by the Epworth Sleepiness Scale (ESS) [18]. However, in asymptomatic patients, the questionnaire method is not recommended for diagnosis because of its low sensitivity in such a case [19].

Although ESS has been commonly used to study daytime sleepiness in hypertensive patients with OSAHS [20, 21], to date little attention has been devoted to the comparison of ESS scores in hypertensive patients and normotensive patients in screening for OSAHS. The objective of this research is, therefore, to compare the ESS score in hypertensive patients with severe OSAHS and that of normotensive patients with severe OSAHS to determine the extent to which the issue of sensitivity of ESS score may determine the effectiveness of OSAHS screening in the case of hypertensive patients.

## Materials and methods

### Population

We conducted a retrospective study on the basis of a database collated from our institution (Toulouse University Hospital, France). We formed two separate groups of patients, the first one consisting of patients suffering from severe hypertensive OSAHS, and the second one, a control group of normotensive patients with severe OSAHS. Both groups are matched in terms of both age and gender.The database contains the following items: an anonymized checklist of patients examined for an OSAHS; the polysomnography data (Apnea Hypopnea Index: AHI and Oxygen Desaturation Index: ODI); the ESS score; the main clinical paraclinical parameters (age, gender, medical history, cardiovascular risk factors, blood pressure in consultation, or in Ambulatory Blood Pressure Measurement – ABPM); and finally when available, treatment in progress. To constitute the two groups of the study, we included all patients aged ≥18 years at the time of fitting, suffering from severe OSAHS (AHI ≥ 30) objectified by sleep recording (polysomnography or ventilatorypolygraphy), and having already benefited from the completion of an ESS by a qualified technician prior to the implementation of a CPAP treatment.

The absence of HT was defined as follows: the absence of HT reported in the medical dossier, the absence of antihypertensive drugs, Systolic Blood Pressure (SBP) < 140 mmHg and Pressure measurements Diastolic Arterial (DBP) < 90 mmHg in consultation. As for the presence of HT, it was defined by the following features: the personal history of HT, the presence of antihypertensive drug in usual therapy or a measurement of SBP ≥ 140 mmHg and/or DBP ≥ 90 mmHg. Overall, the inclusion criteria comprise the following: an age > 18 years, a severe OSAHS defined by an AHI ≥ 30 and a well-defined hypertensive status. We defined a symptomatic patient with an ESS score > 10.

In accordance with European regulation, French retrospective studies from data obtained without monitoring procedure or additional therapy, do not require the approval of ethics committee.

### Statistical analysis

We calculated the necessary total number of patients to be included in each group in order to obtain sufficient power with an alpha risk of 0.05 and a power of 0.8. Based on a previous study^22^ that found out a 20% difference in normotensive and hypertensive symptomatic patients in an OSAHS population ≥ 15, we estimated that the appropriate number of patients to be included in each group at 93 patients. Accordingly, we included the first 100 patients in each group from our database in compliance with the previously defined inclusion criteria, taking into account the age and sex match. We performed our statistical analysis using STATA statistical software, release 11.2 (STATA Corporation, College Station, TX, USA). Continuous variables were compared with Student’s t-test or Mann-Whitney test when their distribution deviated from normality (or when homoscedasticity was rejected) and qualitative variables using Chi2-test or Fisher’s exact test when needed. A two-sided *p* value < 0.05 was considered statistically significant. A logistic regression initially including all statistically significant variables was then used for the co-factor adjustment.

## Results

### Population of the study (Table 1)

A sample population of 200 patients was divided into two groups of hypertensive (*n* = 100) and normotensive patients (*n* = 100). The mean age of the patients in the study was 56 (± 12) years, 65% of the patients were male. These patients were predominantly obese with an average Body Mass Index (BMI) of 34 (± 8) kg/m^2^. Mean SBP and DBP were 131 (± 14)/75 (± 11) mm Hg. The mean ESS score was 10.9 (± 3.3) and 44% of the patients had symptomatic OSAHS (ESS score > 10). The average AHI was 45.1 (± 15.4)/h and ODI was 36.7 (± 20.0)/h. Both groups were comparable in age, sex and BMI. The SBP and DBP were statistically higher in the hypertensive group compared to the normotensive group (SBP: 138 mmHg vs 124 mmHg, *p* < 0.01 and DBP: 80 mmHg vs 70 mmHg, *p* < 0.01). The ESS score was statistically lower in the hypertensive group compared to the normotensive group (10.0 ± 2.9 vs 11.9 ± 3.5, p < 0.01) though the AHI and ODI showed no significant statistical difference (AHI: 45.0 ± 15.3 vs 45.2 ± 20.1, *p* = 0.91 and ODI: 36.2 ± 20.0 and 37.3 ± 20.1, *p* = 0.74). Moreover, there were fewer symptomatic OSAHS patients in the hypertensive group compared to the normotensive group (30% versus 58% *p* < 0.01).

**Table 1:** Comparison between hypertensive patients and non hypertensive patients

Parameters	All patients (*n* = 200)	HT patients (*n* = 100)	non HT patients (*n* = 100)	*p*
Age (years) (mean, SD)	56, 12	57, 11	55, 13	0.24
Men (*n*, %)	130, 65%	65, 65%	65, 65%	1
Body Mass Index (kg/m^2^) (mean, SD)	34, 8	34, 7	34, 8	0.41
Obesity (*n*, %)	126, 63%	64, 64%	62, 62%	0.72
Asthma (*n*, %)	5, 3%	1, 1%	4, 4%	.
Chronic Obstructive Pulmonary Disease (*n*, %)	8, 4%	2, 2%	6, 6%	.
Epworth Sleepiness Scale Score (unity) (mean, SD)	10.9, 3.3	10.0, 2.9	11.9, 3.5	< 0.01
ESS Score > 10 (*n*, %)	88, 44%	30, 30%	58, 58%	< 0.01
AHI(/h) (mean, SD)	45.1, 15.4	45.0, 15.3	45.2, 15,5	0.91
ODI (/h) (mean, SD)	36.7, 20.0	36.2, 20.0	37.3, 20.1	0.74
Systolic Blood Pressure (mm Hg) (mean, SD)	131, 14	138, 14	124, 9	< 0.01
Diastolic Blood Presure (mm Hg) (mean, SD)	75, 11	80, 11	70, 10	< 0.01

*AHI* Apnea Hypopnea Index

*ESS* Epworth Sleepiness Scale

*ODI* Oxygen Desaturation Index

*HT* Hypertensive

### Anti-hypertensive therapy (Table 2)

Table 2 depicts the proportion of therapeutic classes of anti-hypertensive drugs and frequencies of treatment strategies (monotherapy, dual, triple or quadruple therapy) among the hypertensive group. None of the patients of the normotensive group was treated with antihypertensive drug. Of note, in the hypertensive group, there was no significant difference in SBP, DBP, ESS score, AHI or ODI between different treatment classes or strategies.

**Table 2:** Anti-hypertensive drugs and treatment strategies (Hypertensive group)

Parameters	HT patients (*n* = 100)
Anti-hypertensive drugs	
Betablockers	24, 24%
Angiotensin-Converting-Enzyme Inhibitors	35, 35%
Angiotensin II Receptor Antagonists	43, 43%
Calcium Channel Blockers	56, 56%
Thiazide and Thiazide-like Diuretics	62, 62%
Treatment strategies	
Monotherapy	30, 30%
Dual Therapy	27, 27%
Triple Therapy	36, 36%
Quadruple Therapy	7, 7%

*HT patients* hypertensive patients

### Multivariate analysis

In logistic regression, once adjusted for age, gender, the presence of obesity, SBP, DBP, AHI, and ODI, the absence of HT was significantly associated with symptomatic OSAHS (OR = 2.83, 95% CI = [1.298–6.162], *p* = 0.01).

## Discussion

This research compared the ESS score on a sample of patients with severe OSAHS according to their blood pressure status (HT or not).One of our findings is that hypertensive patients have excessive daytime sleepiness, but assessed as being less important by ESS, and so would be less symptomatic than normotensive patients. Moreover, the research shows a striking difference in the positivity of the ESS (ESS score > 10) between our two groups with a significantly higher positive ESS in normotensive patients compared to hypertensive patients (58% versus 30% *p* < 0.01). These data are reinforced by the comparability between our two groups, particularly on the predictors of daytime sleepiness in the OSAHS (Age, sex, BMI, AHI, ODI) [23].

These same findings confirm the results reached by Martynowicz et al. [22] who had conducted a prospective observational controlled study (HT vs non-HT) in 374 patients receiving nocturnal polysomnography. This work showed that in patients with AHI ≥ 15: the ESS score was significantly higher in normotensive patients compared to hypertensive patients (13.80 ± 6.66 versus 9.84 ± 5.56 *p* < 0.05). Martynowicz et al. demonstrated that in a normotensive population, the ESS score was significantly higher in patients with OSAHS and AHI ≥15 versus patients with OSAHS and AHI < 15. By contrast, in the hypertensive sample there was no significant increase in ESS score between patients with AHI ≥ 15 and patients wih AHI < 15. To our knowledge, few other studies have been published in this subject.A study by Mo et al. [24] on risk factors contributing to the development of hypertension in patients with OSAHS did not find any difference on ESS between hypertensive and normotensive patients. However, all OSAHS and not only severe OSAHS patients were included in this work and the two groups (hypertensive vs normotensive patients) were not comparable for respiratory parameters with higher AHI, ODI and lower minimal pulse oxygen saturation in the hypertensive group suggesting more severe OSAHS in the hypertensive group In a study on 411 hypertensive patients with undiagnosed Obstructive Sleep Apnea, Brostrom et al. [25] found that only 37% of patients had an ESS score > 10 in the subgroup of patients with moderate to severe Obstructive Sleep Apnea (*n* = 121). This result is close to ours with only 30% of ESS score > 10 in the hypertensive group suggesting that the sensibility of ESS is too low to screen Obstructive Sleep Apnea in hypertensive patients.

From a pathophysiological point of view, the OSAHS is responsible for apnea-related micro-arousals, leading to hypoxia and daytime sleepiness. In patients with excessive daytime sleepiness, abnormal activation of the autonomic nervous system occurs at night with a decrease in baroreflex sensitivity and vagal tone, which subsequently impacts the body’s hemodynamics, including arterial blood pressure [26, 27]. This relationship between excessive daytime sleepiness and autonomic nervous system could account for the difference between the two groups of our research on the score of the ESS even if the exact mechanism has not yet been fully explained.

By analyzing the positivity of the ESS (ESS score > 10), the number of symptomatic OSAHS is significantly less important in the hypertensive population than in the normotensive one: it might eventually lead to under-screening for OSAHS in the hypertensive population.

Multiple questionnaires are available for the screening of OSAHS. To our knowledge, no previous studies have compared different questionnaires in the hypertensive population. In the general population, a study [28] compared 9 screening questionnaires (Berlin, modified Berlin, STOP, STOP-Bang and OSA50 questionnaires, sleep apnea clinical score, ESS, American Society of Anesthesiologists checklist, and the elbow sign questionnaire). The Sleep Apnea clinical Score and the STOP-Bang questionnaires were found to have the best performances for predicting OSAHS. However, similar studies showed screening questionnaires are equally unsuitable for other populations, such as the Berlin Questionnaire of the ESS for pregnant women [29] or the ESS for patients undergoing for bariatric surgery [30].

The ESS questionnaire subjectively evaluates excessive daytime sleepiness, which is less reliable than an objective assessment of sleepiness, such as the Multiple Sleep Latency Test, or the Maintenance of Wakefulness Test [31]. Unfortunately, these tests are not yet feasible because they are difficult to access and to implement in daily practice. Use of ESS has also limitations: unlike other questionnaires, such as the STOP BANG questionnaire, it does not take into account the presence of hypertension in its predictive criteria to screen for moderate, mild and severe OSAHS [32]. Obviously, the ESS has an even lower sensitivity in hypertensive patients. It follows that the use of such a type of test for assessing excessive daytime sleepiness is not totally suitable for an effective OSAHS screening in hypertensive patients.

The presence of an OSAHS is a resistance factor of HT. OSAHS treatment with CPAP has been the subject of several studies demonstrating the benefit of the use of CPAP for blood pressure control. A meta-analysis [33] from randomized controlled trials and observational studies confirm that CPAP results effectively in a significant decrease in blood pressure. This benefit, underlined in six different studies, consists in the reduction of - 7.2/- 4.99 mmHg for the SBP/DBP of 24 h in ABPM. Taking into account only the randomized controlled trials the gain is - 6.74 / - 5.94 mmHg. It has to be noted that in this analysis there was a correlation between compliance with CPAP and blood pressure control. On the other hand, there was no relationship regardless of the signs of daytime sleepiness to be mentioned.

Hopefully, these results combined with our findings suggest that blood pressure control can be much more effective and efficient if the OSAHS is much more deeply and broadly explored in hypertensive patients. However, this proposed solution raises the issue of the accessibility of diagnostic tests such as polysomnography and the economic issue pertaining to the feasibility of generalizing these tests to an ever-increasing hypertensive population. The use of nocturnal oximetry [34] or the development of a screening test specific to the hypertensive population could be an intermediate solution.

This research has some limitations that can be summarized as follows. Firstly, it is a small monocentric retrospective study with all the limitations due to this type of work. Naturally, it will have to be supplemented by larger retrospective studies. Secondly, there might be a selection bias in our sample. It is also possible OSAHS was tracked in hypertensive patients because of their hypertension rather than symptoms suggestive of OSAHS. However, despite these drawbacks, the comparability of our two groups in terms of predictors of excessive daytime sleepiness in patients with OSAHS (age, gender, BMI, obesity, AHI, ODI) supports our hypothesis.

## Conclusion

In our study on patients suffering from a severe Obstructive Sleep Apnea Hypopnea Syndrome, the Epworth Sleepiness Scale score was lower in hypertensive patients than in normotensive patients. This discrepancy can be accounted for either by a difference in symptoms or by the non-validity of ESS in hypertensive patients and may lead to an underestimation of severe OSAHS in hypertensive patients. The systematic use of polysomnography to a larger number of hypertensive patients could be envisioned, but in no way can a low ESS score should stop the investigation for screening for OSAHS.

## Abbreviations

AASM: American Academy of Sleep Medicine
ABPM: Ambulatory Blood Pressure Measurement
AHI: Apnea Hypopnea Index
BMI: Body Mass Index
CPAP: Continuous Positive Airway Pressure
DBP: Diastolic Blood Pressure
ESS: Epworth Sleepiness Scale
HT: Hypertension
ODI: Oxygen Desaturation Index
OSAHS: Obstructive Sleep Apnea Hypnopnea Syndrome
SBP: Systolic Blood Pressure
